# Common Misconceptions about the Phonological Deficit Theory of Dyslexia

**DOI:** 10.3390/brainsci11111510

**Published:** 2021-11-14

**Authors:** David L. Share

**Affiliations:** Department of Learning Disabilities, Edmond J. Safra Brain Research Center for the Study of Learning Disabilities, University of Haifa, Haifa 3498838, Israel; dshare@edu.haifa.ac.il

**Keywords:** dyslexia, reading, phonological, neurobiology

## Abstract

In this discussion paper, I review a number of common misconceptions about the phonological deficit theory (PDH) of dyslexia. These include the common but mistaken idea that the PDH is simply about phonemic awareness (PA), and, consequently, is a circular “pseudo”-explanation or epiphenomenon of reading difficulties. I argue that PA is only the “tip of the phonological iceberg” and that “deeper” spoken-language phonological impairments among dyslexics appear well before the onset of reading and even at birth. Furthermore, not even reading-specific expressions of phonological deficits—PA or pseudoword naming, can be considered circular if we clearly distinguish between reading *proper*—real meaning-bearing words, or real text, and the mechanisms (subskills) of reading development (such as phonological recoding). I also explain why an understanding of what constitutes an efficient writing system explains why phonology is necessarily a major source of variability in reading ability and hence a core deficit (or at least one core deficit) among struggling readers whether dyslexic or non-dyslexic. I also address the misguided notion that the PDH has now fallen out of favor because most dyslexia researchers have (largely) ceased studying phonological processing. I emphasize that acceptance of the PDH does not imply repudiation of other non-phonological hypotheses because the PDH does not claim to account for all the variance in reading ability/disability. Finally, I ask where neurobiology enters the picture and suggest that researchers need to exercise more caution in drawing their conclusions.

## 1. The Phonological Deficit Hypothesis of Dyslexia: A Scientific Success Story

The Phonological Deficit Hypothesis (PDH) of dyslexia, in many ways, is a model of true scientific progress. This success story began over half of century ago at Haskins Laboratories, New Haven, Connecticut, where unplanned and unanticipated discoveries about the nature of the speech code [1] lead to a new appreciation of the complexities of “extracting” phonemes from the speech stream [2] and the far-reaching implications of these insights with regard to the challenges of learning to read a writing system based on phonemic units [3,4,5]. This was by no means the first time that a child’s difficulty with what today is called “phonological awareness” was mooted by educators and psychologists as a cause of reading problems and dyslexia. Well before the Haskins breakthroughs, many educators had speculated, albeit in rather vague intuitive ways, about the role of “auditory discrimination” or “sound analysis” in learning to read and dyslexia, but lacking the firm theoretical foundation that the Haskins group provided, this issue was poorly understood and often confused with auditory-perceptual acuity. 

Alongside the breakthroughs in phonological awareness, a steady accumulation of convergent findings pointed to subtle sub-clinical abnormalities in preliterate *spoken* language among dyslexics. These signs, also noted decades earlier by physicians and clinicians such as Samuel Orton [6], were later formalized as the diagnostic and research tools widely known today as confrontation (picture) naming, word-finding, short-term phonological memory (e.g., digit span), pseudoword repetition, pseudoname learning, speech perception and production, as well as Rapid Automatized Naming (RAN) (although opinion is still divided as to what extent RAN can be considered “phonological”, see, e.g., [7,8]). Together with phonological awareness, this phonological ensemble was collectively labeled “phonological processing” [9,10,11,12,13] and the new Phonological Deficit Hypothesis of Dyslexia was born, rapidly displacing earlier perceptual and sensorimotor hypotheses of dyslexia and, moreover, spawning a new generation of interventions that proved effective tools for educators and clinicians. The torrent of converging research findings came to the boil in the final decade of the twentieth century with numerous independent reviews and meta-analyses of the research literature (see, e.g., [4,14,15,16,17,18,19]), spilling over into the early years of the 21st century ([20,21,22,23,24,25,26,27]; and countless other papers). 

Today, the PDH remains the status quo to the extent that no behavioral or brain-imaging investigation of dyslexia can afford to neglect the assessment of phonological skills in either the definition or validation of their dyslexic sample. Yet, despite the remarkably broad consensus about phonology, there remain loose ends to be tied up to which I return in my concluding remarks.

As is so often the case with any successful scientific theory that has been popularized well beyond the bounds of the research community, there are inevitable misunderstandings and misconceptions that have gained currency over the years. My purpose here is to address and hopefully dispel some of these misconceptions about the phonological deficit hypothesis of dyslexia.

**Misconception** **1.** *The phonological deficit hypothesis of dyslexia (PDH) is just about deficits in phonemic awareness (PA) and, consequently, is merely a circular “pseudo-explanation” or epiphenomenon of reading difficulties.*

There are actually two misconceptions here. First, the PDH is not just about phonemic awareness (PA): PA is only the tip of the phonological iceberg. Second, even if we consider only the PA deficits among dyslexics, such deficits are not a circular pseudo-explanation or epiphenomenon of their reading difficulties any more than other levels of explanation such as genes, neurobiology, or poor teaching.

I begin with the most common misunderstanding about the PDH—that the PDH refers solely to deficits in PA.

*Deficits in PA are only the tip of the phonological iceberg, and like other reading-specific subskills such as pseudoword reading and letter knowledge, is a genuine (proximal) explanation of poor (word) reading.*

Phonology is far more than just phonemic awareness. (Throughout this paper, I refer only to *phonemic* awareness rather than the more general topic of “phonological awareness”, which typically covers a broader array of phonological units from single phonemes, through multi-phonemic sub-syllabic units such as rimes and bodies, to whole syllables. This is because almost all of the research literature focuses on the awareness of phonemes rather than other “larger” units. Furthermore, the role of larger supra-phonemic units remains controversial, especially when the question of dyslexia in languages other than English arises.) Like other reading-specific subskills such as letter knowledge and pseudoword naming, PA is often the product of “deeper” spoken-language phonological weaknesses that predate literacy acquisition [28] and, indeed, go right back to birth [29,30,31]. Ever since the dawn of the dyslexia concept, physicians and clinicians have noted subtle sub-clinical “signs” of spoken-language abnormalities primarily (but not exclusively) in speech processing among dyslexics (e.g., [6]). These signs are not obvious because dyslexics are, to a large extent, competent speakers of their native tongue. However, because phonology is so crucial for learning to read, as I elaborate below, they only become “visible” in literate societies. (This is one reason why some have claimed that dyslexia is a modern “invention”—a myth designed to blame the child for poor teaching.) The classic dyslexic signs include word-finding and word-retrieval problems, difficulties recalling addresses, telephone numbers, the days of the week or months of the year, foreign names and places, early speech production and later articulation difficulties especially with phonologically challenging material such as tongue-twisters. All of these tasks are especially taxing for phonological processing (input and output) because relatively abstract verbal labels have little or no inherently meaningful referent to help compensate, and, on occasion, circumvent the phonological weaknesses. Ironically, many of these everyday “tasks” are all modern literate inventions, and would not have “exposed” a dyslexic profile in a pre-literate world. Few would have noticed such subtle weaknesses in oral language except perhaps when memorizing and reciting prayers or scriptures, especially in sacred and unspoken languages such as Latin, Sanskrit or Arabic.

Once we take into account the full spoken-language and written-language phonological picture, and not merely a short-sighted, truncated PA-only view of the PDH, the notion that the PDH is merely a circular explanation of reading disability/dyslexia can be dismissed: We have another more “distal” non-reading and pre-reading yet phonological “level” of explanation. (Coltheart and Jackson [32] proposed a very useful distinction between “proximal” and “distal” causes of reading difficulty, *“… in considering the causes of reading difficulty it is essential to distinguish between proximal cause (some abnormality in the information-processing system that the child is using to read) and distal cause (the reason for this system being abnormal). The same proximal cause (e.g. poor phonic knowledge) can be the product of different distal causes in different children (e.g. the distal cause might be constitutional, environmental, or educational).” P. 1.* I adopt the proximal-distal terminology but avoid the deterministic term “cause” preferring more probabilistic terms such as “precursors” (or risk factors) which are more consistent with the current research consensus.)

Returning to the tip of the iceberg analogy, it is clear that once we look below the surface (i.e., beyond PA) we see that, this ensemble of phonological deficits are true precursors or antecedents of reading disability/dyslexia in both a temporal and logical sense.

### 1.1. Why the PA-Only Shortsightedness?

There appear to be several reasons why the spoken-language roots of phonology are so often ignored when discussing the PDH. First, phonemic awareness has monopolized the attention of most psychologists, educators, and neuroscientists for multiple reasons. (1) The correlations between PA and tests of word reading are typically among the strongest (e.g., [33]), (2) PA is readily teachable, and (3) experimental training studies (e.g., [20,34,35]); have demonstrated a powerful influence of PA on early reading progress (at least in reading accuracy). In contrast, attempts to “remediate” other aspects of spoken-language phonological weaknesses have not met with much success and consequently have little to offer the educator or clinician. As a result, PA has received immense publicity in reading education circles and even in public debate on the teaching of reading, especially in the US and the UK [21,36].

Yet, another reason why dyslexia researchers have been preoccupied with PA and ignored other aspects of phonology may be the growing popularity of the Double Deficit Hypothesis of dyslexia [37], which has also (unwittingly) helped promote the view that phonological deficits are just about PA, and that rapid serial naming (RAN), which has traditionally been classified under the rubric of phonological processing [9,10,13], represents a separate, additional, and unique source of difficulty among dyslexics.

However, even when spoken-language phonological deficits are overlooked, there is nothing circular about PA in and of itself as a true *proximal* explanation of dyslexia, provided we are careful to distinguish between reading proper and the mechanisms or subskills necessary for reading to develop.

**Misconception** **2.** *Deficits in PA (and other reading-specific skills such as pseudoword naming) are circular or pseudo-explanations of reading difficulties.*

Even though phonemic awareness, like letter knowledge and pseudoword naming, is an essential component subskill of an efficient reading system and hence reading-specific, this does not mean that PA is a circular explanation or epiphenomenon of reading difficulties. However, I first wish to emphasize that phonemic awareness is not an emergent linguistic capability.

The evidence is clear that awareness of *phonemes* develops only in the context of the development of *alphabetic* literacy and does not appear to be an integral part of humans’ biological preparedness for rapid, early, universal, and seemingly spontaneous spoken language acquisition. In the words of Bowey [38], phonemic awareness is “inextricably linked” to learning to read an *alphabetic* orthography (p. 168). Additionally, unlike spoken language competencies, reading is a skill that requires a conscious investment of time and effort, explicit instruction and a great deal (years) of practice. Moreover, as an integral component of alphabetic literacy learning, phonemic awareness is closely tied to the particular orthography being learned, the fidelity of the spelling–sound mappings, the timing of alphabetic *and only alphabetic* reading instruction and even the type of instruction (see Share, [39], pp. 596–599, for more detailed discussion). Children who are taught to read whole syllables but not explicitly taught the phonemic principle (that letters or the quasi-syllabic aksharas in Indic scripts) map phonemes do not spontaneously develop awareness of phonemes [40,41,42]. Like learning to read (English) which Gough famously dubbed “unnatural” [43], see also [3], becoming aware of the constituent phonemes in spoken words does not come “naturally”. The available evidence therefore, converges on the conclusion that phonemic awareness is not a universal and emergent linguistic capability [10,39] but is best categorized as a reading *subskill*.

If phonemic awareness is reading-specific, then a truncated PA-only version of the PDH that equates to deficits in phonemic awareness alone begins to look like a circular explanation (pseudo-explanation) of reading difficulties. This is incorrect not only for the reason discussed above (PA is just the tip of the iceberg) but for another equally compelling reason.

*Reading disability/dyslexia cannot be defined on the basis of either PA, pseudoword naming or any other reading subskill.*

On both sides of the Atlantic, current definitions of dyslexia unambiguously specify ***word*** reading, that is, the reading of ***real*** (typically isolated) words.

*“Dyslexia is evident when accurate and fluent **word reading** and/or spelling develops very incompletely or with great difficulty”,*

(British Psychological Society [44])

*“Inaccurate or slow and effortful **word reading** (e.g., reads single words aloud incorrectly or slowly and hesitantly, frequently guesses words, has difficulty sounding out words”*.

(p. 84). DSM-5 [45]

In Europe, too, benchmark measures of reading ability (and disability/dyslexia) (e.g., the Alouette Test in French [46], or the Een Minuut (One Minute) Test in Dutch [47]) all typically employ *real* words and real words alone. Researchers must not lose sight of the purpose of reading, which is accessing *meaning*—at the level of single words, phrases, sentences, and text—because reading is a purposeful socio-cultural practice, and in today’s literate societies the ultimate goal, indeed the very raison-être of compulsory schooling, is becoming literate (and numerate), namely, understanding, learning from, reflecting on, producing and enjoying written language. (Historically, this was not always the case [48].) Some educators would even argue that reading isolated (real) words is already one step removed from “real” reading which is “connected text”, but a few moments spent in a supermarket, a pharmacy, on the road, or on the internet shows that reading in the “real world” includes many single words too.

This means that benchmark tests for defining reading skill and reading disability/dyslexia cannot be based on pseudoword naming or PA and can only be based on real words, real sentences and real texts. If a child presents difficulties on such benchmark measures, then it makes sense to dig “deeper” to uncover the reasons. No competent reading specialist or diagnostician these days would neglect to assess pseudoword reading or PA, but in the classroom the *purpose* of teaching children to read (and write) is not fast and accurate pseudoword naming or phonemic segmentation. (Of course, the question of assessing pre-school “readiness” for reading is different and here it behooves educators to assess letter knowledge and PA—the two key co-determinants [38] or co-requisites [10] of early reading.)

PA, like other reading subskills such as phonological recoding (decoding) and letter knowledge are co-requisites [10] or co-determinants [38] of reading development, **but are not reading per se** in the sense of purposeful socio-cultural practice. Of course, just knowing that a child has poor PA or pseudoword naming says nothing about the “distal” source of this proximal obstacle to reading. If, following Coltheart and Jackson [32], we make a clear distinction between reading per se and the requisite “mechanisms” or sub-skills of reading, then we can say that poor phonological recoding (decoding) skills (manifest in poor pseudoword naming) or poor phonological awareness are “true” proximal explanations of poor (word) reading. (Half a century ago, prior to the ascendance of the PDH, the “proximal” causes of poor word reading were sought elsewhere in perceptual processing such as letter discrimination and visual processing (e.g., [49,50]). If, however, we conflate the goal and the means to achieve this goal then, and only then, do we risk accusations of circularity and pseudo-explanations of reading failure.

Needless to say, neither poor PA nor slow and/or inaccurate word reading or pseudoword reading can distinguish between biologically based reading difficulties (i.e., classic IQ-discrepancy defined dyslexia) and cases of reading difficulty stemming from non-biological environmental and/or instructional factors. Distinguishing between what some researchers like to call “hard-core” or “true” dyslexia as opposed to non-dyslexic poor readers was the logic of the Response to Intervention approach (RTI) pioneered by Vellutino and colleagues [51] that has now become the norm in the US. If a child who has not had an adequate opportunity to learn to read (poverty, adverse home circumstances, inadequate or insufficient instruction, etc.) is given good evidence-based instruction (either in whole-class settings, or, failing that, in more intensive, small-group instruction), and still fails, then we can conclude that the source of the difficulty is probably an intrinsic neurobiologically based dysfunction (“constitutional”) that calls for more intensive one-on-one daily instruction with a specialist.

*In summary, there is nothing circular or trivial about poor PA “explaining” dyslexia (at the **proximal** behavioral level of analysis). On the contrary, this has been one of the success stories of modern reading science.*

**Misconception** **3.** *Most dyslexia researchers today have ceased studying phonological processing. Doesn’t this show that the PDH has fallen out of favor?*

Where are the studies on phonology today? There aren’t any because we don’t need to re-invent the phonology wheel. Today’s research establishment has accepted, indeed institutionalized the role of phonology in reading. Researchers today are addressing at new beyond-phonology questions searching for the roots of the phonological deficit—at the neurobiological level—looking for additional non-phonological sources of difficulty, and looking at other languages and writing systems, but no self-respecting journal today would consider publishing a study reporting deficits among dyslexics in pseudoword reading or PA (at least not in English), because there is nothing new or disputed here. Science concerns itself with tackling *unresolved* questions with the goal of creating new knowledge or refining existing knowledge. Journals are not interested in publishing “old news”.

Summing up, the role of phonology is reading and dyslexia is almost universally taken for granted today, perhaps almost trivially obvious, but what today seems obvious (like many successful advances in science that topple the old status quo and become well-established “facts”), was the product of decades of empirical and theoretical work that had to prove itself against a very different status quo.

*Why phonology is necessarily a major source of variability in reading ability and hence a core deficit (or at least one core deficit) among struggling readers whether dyslexic or non-dyslexic.*

Phonology is an inescapable feature of writing system architecture. No writing system can function effectively without it. Every writing system incorporates both symbols that represent the sounds of a specific language (phonology) as well as meaning (morphology). This is true of every extant writing system today including Chinese [52,53], and is even true of the very earliest forms of writing—ancient Chinese, Egyptian hieroglyphics, Mayan, and Sumerian. Phonology is a *sine qua non* of writing (and of reading [10,24]) simply because an efficient writing system cannot function without serving the needs of the novice for *decipherability* (the ability to decode new words) and *learnability* and this can only be achieved via a workable degree of phonological transparency—systematic or semi-systematic correspondences between symbols and sound. It is equally important to note that an effective writing system cannot rely solely on phonology (hence phonology is not the whole story of reading ability and disability/dyslexia, as discussed below), because it must also fulfill a second function—addressing the needs of the expert-to-be for *unitizability*—necessary for rapid, whole-word (or whole-morpheme) recognition via *morphemic transparency*—systematic or semi-systematic correspondences between symbols and meaning (e.g., the English plural marker ⟨s⟩ or the past tense suffix ⟨ed⟩; [52,53,54]). The idea that children learn how to combine or chunk (unitize) meaningless groups of elements such as letters into meaningful higher-order units (morphemes and words) has featured in almost every English-language theory of learning to read proposed over the past half century [55,56,57,58,59,60,61]. Here, I confine my comments to the decipherability/learnability criterion (elsewhere [53], I present a more detailed discussion of the morphological criterion).

Because all printed words are unfamiliar at some point in development (even a child’s own name), the beginning reader needs a means of identifying new words. Neither contextual guessing, nor direct instruction affords a feasible means of unlocking word identities [9,10,39], so the child needs some means of *independently* identifying novel words. This is achieved mainly (but not solely) by systematic or quasi-systematic mappings between the graphic symbols and units of the spoken language. In morpho-syllabic writing systems of which only Chinese survives today, semantic cues (available from the semantic radical) are combined with phonological cues (the phonetic) to allow children to identity (many) novel words by themselves at the intersection of meaning (including context) and sound [62] Although English is a fundamentally alphabetic script, it also has a strong morphemic element [63,64,65] (consider the past tense suffix ⟨ed⟩) that is also helpful in identifying novel words—their meanings and pronunciations). English is not easily decipherable as the evidence clearly shows [39,66,67] but it is *sufficiently* regular (mainly in the consonant letters which are more informative about word identity than the fickle vowel letters).

### 1.2. Decipherability, Learnability and Combinatoriality

Decipherability is possible by virtue of the *combinatoriality* of writing systems—this refers to the fact that a limited (and hence learnable) number of elements (alphabetic letters, Indic aksharas, Japanese kana, etc.) can be combined and recombined to generate an unlimited number of units of meaning—morphemes and words. This combinatoriality—infinite ends from finite means, is a fundamental design principle of all languages whether spoken [3,68,69,70], written [53] or signed [71,72]. In spoken language, a few dozen phonemes can be combined to generate thousands or tens of thousands of morphemes, which can further be combined into an infinite number of possible words in a nested hierarchy of increasingly complex units. (This same combinatorial or “particulate” principle common to all human language (spoken, written or signed) can also be found in many hierarchical systems including chemistry and music [73,74]). Likewise, a writing system is workable if and only if it is based on a *limited* and *learnable* number of sub-lexical graphic units (letters, aksharas, kana, etc.) that can be combined into a potentially infinite number of meaning units. These “elementary particles” or building blocks of writing can only be phonological units (not units of meaning) and must be sub-lexical, that is, below the level of whole words, such as phonemes, sub-syllabic units such as core syllables [75], or, if the syllable inventory is relatively small, whole syllables as in Japanese kana [76].

In the absence of combinatoriality, as in the mythical case of a pure logography, each word or morpheme must be learned anew and memorized as best as possible with the aid of mnemonics. Without phonology, the curious pre-literate child as yet unaware of the combinatoriality of print, resorts to “logographic reading” [56,59,60,61,77], epitomized in attempts to read, or write by looking for referential elements such as the two “eyes” in the word ⟨look⟩, the “tail” at the end of the word ⟨dog⟩ or producing (in the case of writing) enlarged letters for writing ⟨elephant⟩, or using the color red for ⟨tomato⟩. Almost immediately, however, this strategy runs into insurmountable difficulties generating unique “clues” for each word, which rapidly leads the learner up a blind and very short alley [56,57,78,79,80]. This non-systematic, non-combinatorial rote learning is little different from trying to memorize thousands of phone numbers—a feat few are capable of, yet this is precisely the type of learning that is often and erroneously claimed to be how Chinese children learn to read! [27]. These observations explain why no writing system, even Chinese, contains more than a handful of pictograms (e.g., 木 ‘tree’) or ideograms (e.g., 二 ‘two’) that directly convey meaning [77,81,82].

This means that the reader of *any* script must come to appreciate the combinatorial infrastructure of the orthography s/he is learning—how the basic building blocks of writing map into the speech code—and this can only be achieved at the sub-lexical level [76]. In addition to learning the specific symbol-sound mappings of the orthography being learned, the learner must “get inside words”, go below the level of meaning and penetrate their sound structure. This phonological analysis or “meta-linguistic” awareness is an inescapable pre-requisite for literacy learning enabling the learner to exploit the combinatoriality of writing, decipher novel letter strings, match up spellings and pronunciations, and begin the process of building the orthographic lexicon by unitizing or chunking sub-lexical symbols into higher-order meaning units—the key to rapid automatic word recognition. It follows that any difficulties that a novice reader may have in processing speech sounds (e.g., hearing loss) or difficulties (in the absence of hearing impairment)) in processing the nuances of phonology (speech sound disorder, dyslexia) will almost invariably impair learning to read. Here, the evidence is incontrovertible and goes well beyond phonological awareness to early pre-literate spoken language competencies in processing (receptive and expressive) the sounds of speech as discussed earlier. **Phonology, therefore, is *necessarily* a major source of variability in reading ability and hence a core deficit (or at least one core deficit) among struggling readers whether dyslexic or non-dyslexic.**

However, phonology alone does not and cannot explain *all* the variability in reading [83] because as hinted above, meaning units (morphology) must also be represented and clearly demarcated in a writing system. The unitization and automatization of sets of letters into integrated chunks (here written language mimics spoken language) that can be rapidly and efficiently recognized principally at the level of morphemes is facilitated by various demarcation devices in writing such as word spacing (in English), character size uniformity (in Chinese) morpheme demarcation within poly-morphemic words (in Finnish for beginning readers), word-initial capitalization (German, and, in former times, English too), uniquely word-final characters (e.g., Thai, and Arabic), all of which help the reader chunk groups of symbols (letters, aksharas, kana, characters – the minimal units of writing) and without which, reading speed is significantly impaired [84,85]. This means that, contrary to popular belief, the phonological principle is not the sole basis of all reading, or even learning to read.

This analysis highlights another common misconception about the PDH.

**Misconception** **4.** *Acceptance of the PDH necessarily implies repudiation of other (non-phonological) hypotheses.*

The PDH does not purport to explain all the variance in reading ability or, by corollary, explain all the difficulties experienced by all disabled readers or dyslexics [83]. It is not a unitary deficit hypothesis. Clearly, there are other sources of variance in reading ability. (I share the contemporary view that dyslexia is a dimensional, not categorical disorder, that is, it is not a distinct category of readers [86], but merely the lower end of a continuum of severity [87,88] and many more.) There are other candidates at the *behavioral* level of analysis such as visual attention (Facoetti), Visual Attention Span (Valdois), procedural learning (Nicolson & Fawcett), but to date, none of these additional candidate (or competing) sources of variance has remotely approached the consensus achieved by the PDH. In most cases, a single researcher or team has compiled an impressive body of evidence in support of their own theory, but other independent teams have either failed to replicate the empirical findings with additional controls, or have replicated the findings but offered a different interpretation (see for example, [7,8,89,90,91] with respect to the visual attention span (VAS) hypothesis).

Another proposal regarding sources of variance other than PA has been put forward by Wolf and Bowers [37] who have made a strong case that Rapid Automatized Naming (RAN) represents an additional unique source of variance in reading which cannot solely be regarded as a member of the phonological processing family as traditionally claimed [9,10,13]. According to the Double-Deficit Hypothesis (DDH) of dyslexia, dyslexics can be subdivided into three varieties, or subtypes; a group with selective deficits in PA, a group with selective deficits in RAN, and a “double-deficit” subgroup with both PA and RAN deficits who are the most severely disabled readers. The DDH has generated a great deal of controversy in the research literature [7,92,93], but, more recently, a growing (and more favorable) interest among researchers studying dyslexia in more transparent orthographies [94,95,96].

*Summing up, phonology is not, indeed cannot be the whole story to reading ability and disability/dyslexia*.

## 2. Where Does Neurobiology Enter the Picture?

Neurobiology (and genetics) supplies another link in the multi-level chain of explanation, but both are more “distal” levels of explanation. Unfortunately, neurobiological hypotheses of dyslexia are often erroneously interpreted as the “real” causes of dyslexia partly because post-mortem and in vivo brain imaging evidence appears to offer tangible evidence of pathology, (unlike most psychological constructs). The appeal is very seductive—we can literally see the heterotopic malformations in Galaburda et al.’s post-mortem cortical sections of dyslexic brains [97], we can virtually “see” the (computer-visualized) disconnections in the sub-cortical white matter tracts of dyslexic brains, and the smaller and “cooler-colored” “blobs” of under-activation of brain regions using fMRI. This work has, understandably provided a tremendous fillip for the much-debated and still what many (at least in educational circles) regard as the questionable notion of dyslexia as a neurobiological condition. However, so too did the Response to Intervention (RTI) movement that provides another way to separate presumably biologically based dyslexia from instructional-environmental factors that are also extremely potent sources of reading difficulties [26,98]. However, much of the neurobiological hype still rides on the wave of opinion that infers from the brain evidence that we are seeing the real “hard” or “true” *causes* of dyslexia and in so doing overlooks the role of neuroplasticity: the fact that the brain is an organ like any other in the body that matures, changes, and atrophies depending on its use, misuse or disuse. It is not, as was once believed to be the case with IQ, something fixed and innate (which helped promote the now discredited IQ-discrepancy definition of dyslexia). Similar to the well-known *correlation is causation* fallacy, any neurobiological differences between dyslexics and controls may be the product, and not necessarily the cause of the reading problem. The evidence now documents how literacy learning changes our brain [99,100]. David Olson’s [101] World on Paper has now become the World on Screen as print and the ubiquitous 2D screen now permeates almost every moment of our waking lives, reshaping language, our perceptions of language (not just phonology), cognition and even visual processing of the non-print visual world [99]. This means that once literacy is acquired (or failed to be acquired) associations between almost any behavioral “deficit” or brain “signature”—functional or anatomical—are, given the reality of neuroplasticity, just as likely to be a *consequence* of reading failure as a *cause:* correlation is not causation.

The challenge for the neurobiology of dyslexia, therefore, is to establish that any putative neurobiological signature—temporal (Tallal), magnocellular (Stein), cerebellar/proceduralization (Nicolson & Fawcett), ciliary (Kere), oscillatory (Pugh & Hoeft), anchoring (Ahissar), asynchrony (Breznitz) (i) *predates* the onset of reading, (ii) *predicts* future reading outcomes (or at least significant variance) and, especially important (iii) *coheres and converges* with current knowledge about the reading process and, inter alia, the role of phonology. This means that any neurobiological account of dyslexia (like any behavioral hypothesis) needs to be clear about where it stands vis-à-vis the PDH. Many new behavioral and neurobiological accounts often present data from groups or individuals with dyslexia without specifying whether the new hypothesis is (i) ***instead of***, (ii) ***in addition to***, or (iii) ***underpins*** phonology.

An example of an explanation of dyslexia that has been claimed to ***underpin phonological deficits*** is Tallal’s temporal processing hypothesis [102,103,104]. This account converges well with the PDH because sensitivity to rapid acoustic changes in speech would be expected to impair aspects of speech processing important for the fine-grained phonological representations on which tasks assessing phonological processing are assumed to depend.

The visual attention span (VAS) hypothesis advanced by Valdois [105] acknowledges the importance of phonology, but proposes that VAS deficits exist ***in addition*** to and independently of phonological deficits. That is, these deficits represent another distinctly non-phonological core deficit. A similar claim has been made by Wolf and Bowers [37] regarding the contribution of RAN to dyslexia in the context of their Double-Deficit Hypothesis.

Finally, there are (more rarely) those who propose explanatory models of dyslexia that discard or replace the PDH. For example, Vidyasagar and Pammer’s [106] visuo-spatial attention account of dyslexia explicitly rejects the PDH, claiming that their account replaces the PDH.

Summarizing, neurobiology cannot be seen as *the* basis, source or real cause of dyslexia any more than any other “level of explanation” whether this be PA, the DCDC2 gene or well-trained teachers delivering quality reading instruction. It is ***one link in an interlocking multi-level explanatory chain*** extending from genetic risk factors through neurobiology, cognition and language, to environmental-instructional factors including the home, schooling, and the broader socio-cultural-historical setting, all of which interact with brain plasticity. Neurobiological accounts must connect coherently to the available evidence at other levels of explanation [107].

## 3. Summary and Conclusions

Although the PDH, and the role of phonology in reading in general is a model scientific success story, one that allied fields are striving to emulate, there are still loose ends to be tied up and limitations on the explanatory power of phonology. For example, the benefits of PA training are not uniform across all aspects of word reading—accuracy, rate and fluency—and these qualifications have major implications for dyslexia assessment, diagnosis and intervention (see, e.g., [37,108,109]). And the search continues for other non-phonological sources of variation in reading ability and disability/dyslexia because phonology is not the whole story. 

It must also be admitted that this success story has largely been an Anglophone affair, or at least an Anglo-European affair, hence a growing interest in the role of phonology in other languages and orthographies [110,111,112,113]. This fine-tuning notwithstanding, there is no longer any need to re-invent the phonological wheel; “old news” will not make headlines, researchers need to press on to new unresolved questions—what lies beyond and beneath phonology? The advent of brain imaging technologies, and more recently, the RTI movement, brought new hope and a great deal of public anticipation that the long dreamed of biological (and genetic) basis of “true” developmental dyslexia was in our sights. There have unquestionably been genuine breakthroughs in the understanding the neurobiological circuitry of reading and dyslexia. However, the initial hype that neuroimaging would soon uncover the true “cause” of dyslexia has since been tempered by an appreciation of neuroplasticity—the brain became a dependent (and not only independent) variable and no longer a constant in the dyslexia equation. Today, neurobiologists can no longer present their findings in a vacuum, as if this level of analysis enjoys explanatory precedence over all other levels. Behavior, cognition, neurobiology, genetics, environmental-instructional, and socio-cultural-historical factors all have an important role to play as *complementary* not competing levels of explanation in an interlocking multi-level account of dyslexia. Too often neurobiologists fail to consider how their new hypothesis intersects, or fails to intersect with what is known at other levels of analysis. Should the new hypothesis be seen as an ***addition to*** what is already known, ***underpinning*** phonology, or ***instead of***, that is, ***replacing*** the PDH? Or some combination of these possibilities? Above all, scientific progress requires convergent findings from independent teams of researchers. And, it is precisely this—consistent and repeated independent replication—that has been the secret of the PDH success story.
